# Acute Inflammatory Ascites Complicating Clostridium difficile Colitis

**DOI:** 10.7759/cureus.85685

**Published:** 2025-06-10

**Authors:** George S Zacharia, Shivani Jani, Manjola Doda, Satyam Mahaju, Neelanjana Pandey, Harish Patel

**Affiliations:** 1 Internal Medicine, BronxCare Health System, New York, USA; 2 Gastroenterology, BronxCare Health System, New York, USA

**Keywords:** ascites, clostridium difficle infection, colitis, diarrhea, serum-ascites albumin gradient

## Abstract

*Clostridium difficile* (CD) is a spore-forming, Gram-positive anaerobic bacillus that causes toxin-mediated mucosal injury leading to pseudomembranous colitis, clinically characterized by diarrheal disease. Ascites is an infrequent manifestation in severe or fulminant CD colitis. The pathogenesis of ascites in CD colitis is poorly understood but includes hypoalbuminemia due to protein-losing enteropathy, transmural inflammation, toxin-mediated capillary leak, colonic perforation, and concomitant diseases. We report the case of a middle-aged woman who presented with an opioid overdose and subsequently developed severe CD colitis. The infection was complicated with low serum-ascites albumin gradient (SAAG), high protein, culture-negative, neutrophil-predominant ascites, devoid of visceral perforation, or an alternative etiology for ascites. Treatment with oral vancomycin and intravenous metronidazole led to the complete resolution of symptoms and ascites. This case highlights an uncommon presentation of a common healthcare-associated infection and reinforces the importance of recognizing atypical manifestations of CD. While there are no specific management guidelines for this subset, treating the underlying colitis appears sufficient to resolve the ascites in most cases.

## Introduction

Ascites refers to the pathological collection of free fluid in the peritoneal cavity. The etiology of ascites is exhaustive, but the most frequent reason is cirrhosis [1]. Other causes include cardiac failure, hypoalbuminemia, peritonitis, peritoneal carcinomatosis, lymphomas, and many more. Frequently, the peritoneal fluid is non-inflammatory and non-infected. Bacterial infections of ascitic fluid are classified into primary and secondary bacterial peritonitis based on the absence or presence of an intra-abdominal source of infection [2].

*Clostridium difficile* (CD) infections currently rank among the top hospital-acquired infections in the United States [3]. Caused by the Gram-positive, spore-forming, anaerobic bacillus CD, the infection is historically known for its severe toxin-mediated diarrheal disease, association with antibiotic therapy, and high risk of recurrence. The bacteria generate exotoxins, including toxin A (TcdA), toxin B (TcdB), and the binary toxin (CDT). Toxins A and B have been extensively evaluated regarding their pathogenic role in CD colitis and are considered primary virulence factors, potent enterotoxins with additional proinflammatory activities. The toxins, by glucosylation of Rho proteins, cause disruption of the actin cytoskeleton, cell rounding, and death [4]. The loss of colonocytes and tight junctions result in increased mucosal permeability and watery diarrhea. The toxins also induce a massive efflux of proinflammatory cytokines, including tumor necrosis factor α, interleukin 8, and leukotriene B4, from the affected cells and tissue macrophages, resulting in local and systemic neutrophilia, microabscesses, and pseudomembranes rich in neutrophils [5].

Diarrheal disease is the clinical hallmark of CD infection. CD is not a frequent cause of ascites in the pathogenesis of ascites; however, the published literature reports a high incidence of ascites in patients with fulminant infections. The CD is a Gram-positive, spore-forming bacterium that can result in the severe hospital-acquired infection "pseudomembranous colitis" or CD colitis [6]. The retrospective study by Sailhamer et al. reported the presence of ascites on computerized tomography (CT) in 52.7% of patients with fulminant CD colitis. More interestingly, peritoneal free fluid was second only to colonic wall thickening as the most frequent CT finding in fulminant CD infection [7]. The mechanism of ascites is unclear; postulates include hypoalbuminemia due to gastrointestinal protein loss, inflammatory cytokine-mediated capillary leak, transmural colonic inflammation, and micro- or macro-perforations [8]. There are no specific guidelines for managing ascites in CD infection beyond the recommendations for colitis.

## Case presentation

**Figure 1:**
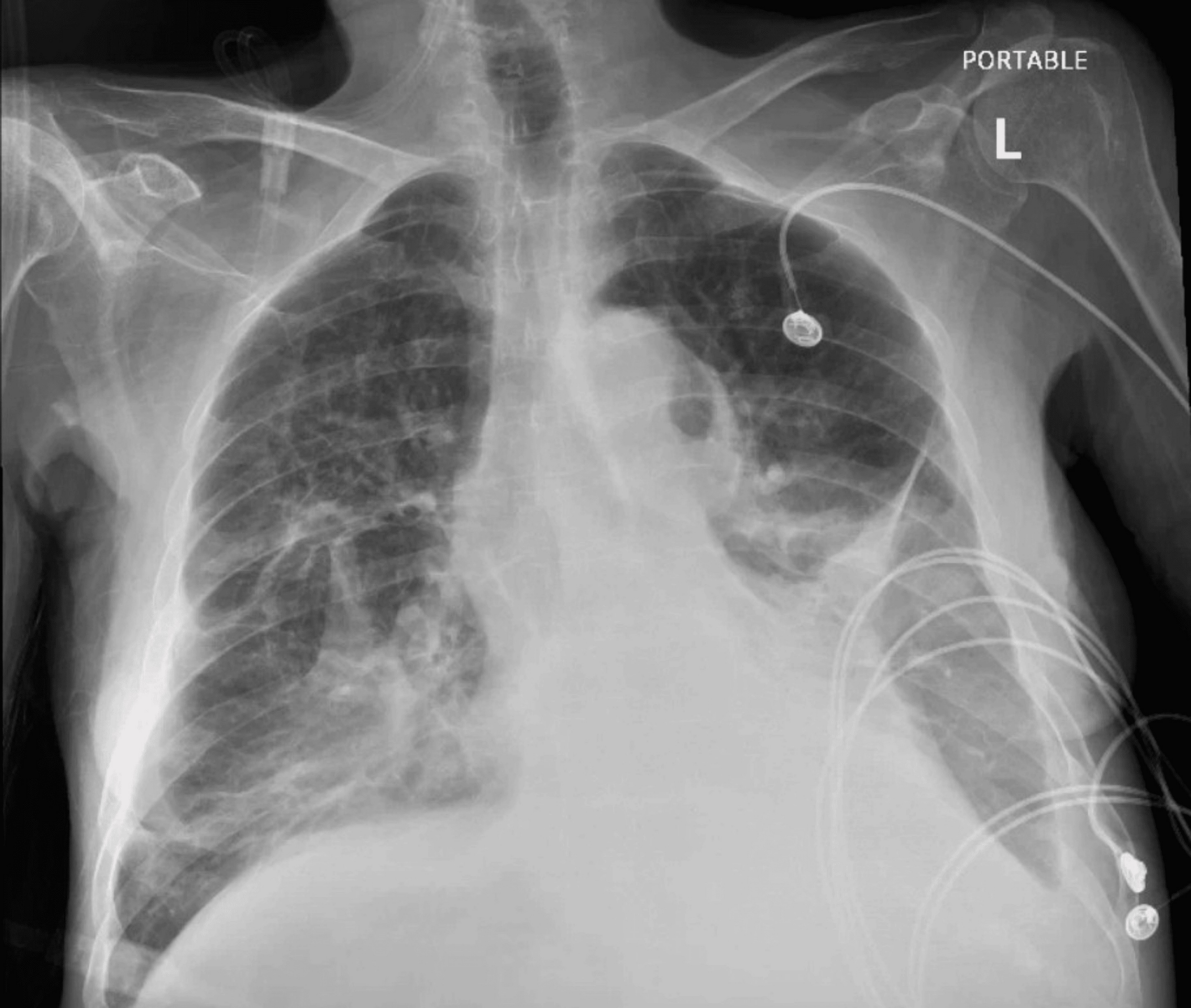
X-ray chest demonstrating bilateral predominantly lower zone opacities

This 69-year-old Hispanic female was brought to the emergency department after being found unconscious at home following an opiate overdose. She has a history of chronic obstructive pulmonary disease, hypothyroidism, and opiate use disorder, with a recent hospitalization four weeks back for exacerbation of lung disease. She received ceftriaxone and azithromycin for three days during the recent hospitalization and was discharged on cefpodoxime for an additional two days for suspected pneumonia. The initial laboratory panel reported anemia, leukocytosis, lactic acidosis, and elevated inflammatory markers (Table 1). A chest X-ray revealed infiltrates involving the bilateral lower lung zones, suggestive of pneumonia (Figure 1). She was initiated on intravenous antibiotics: vancomycin, aztreonam, and azithromycin, in addition to other symptomatic and supportive measures. On the third day of admission, she reported multiple episodes of loose, watery, non-bloody diarrhea and abdominal cramps. Physical examination revealed tachycardia, abdominal fullness with no appreciable shifting dullness, diffuse tenderness, and hyperactive bowel sounds. Stool sample analysis revealed inflammatory diarrhea, with positive results for CD glutamate dehydrogenase (GDH) and toxins A and B. She was initiated on oral vancomycin, intravenous hydration, isolation per institutional protocol, and other supportive and symptomatic measures.

**Table 1:** Summary of hematological and biochemical workup

Parameter	Results					Reference range
	Day 0	Day 3	Day 5	Day 10	Day 14	
Hemoglobin	11.7	13.5	11.5	12	10.9	12-16 g/dL
Mean corpuscular volume	89	89.6	88.4	90.7	88.9	80-96 fL
Leukocyte count	11.2	27.8	42.4	8.2	6.2	4.8-10.8 k/μL
Platelets	323	422	378	290	237	150-400 k/μL
Sodium/potassium	139/4.5	134/4.9	136/4.5	135/4.7	137/4.3	135-145/3.5-5 mEq/L
Blood urea nitrogen	18	22	25	13	17	7-20 mg/dL
Creatinine	1.1	1.4	1.7	0.7	0.5	0.5-1.5 mg/dL
Bilirubin total/direct	0.3/0.1	0.2/<0.2	0.2/<0.2	--	--	0.2-11/0-0.3 mg/dL
Aspartate aminotransferase	18	22	16	--	--	9-36 U/L
Alanine aminotransferase	8	9	6	--	--	5-40 U/L
Alkaline phosphatase	76	73	55	--	--	43-160 U/L
Albumin	2.5	2.3	2.4	--	--	3.5-5.5 g/dL
C-reactive protein	33.9	47.4	69	--	12	<5 mg/dL
Iron	64	--	--	--	--	65-175 μg/dL
Ferritin	196	--	--	--	--	13-150 μg/L
Unsaturated iron binding capacity	108	--	--	--	--	112-346 μg/dL

**Figure 2:**
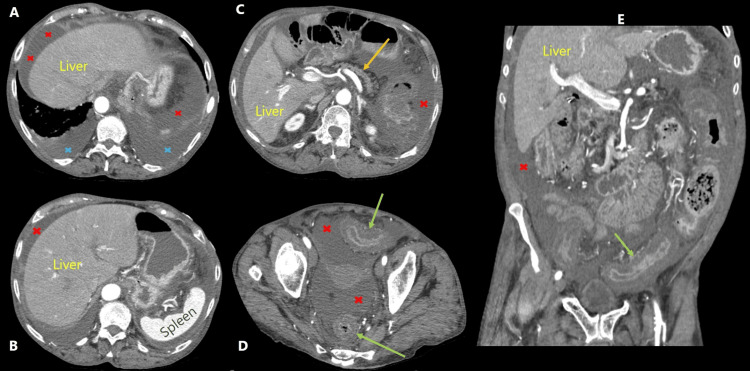
Contrast-enhanced computed tomography images (A-D) Axial images demonstrating ascites (red x), pleural effusion (blue x), a normal-appearing pancreas (yellow arrow), and thickened sigmoid and rectum (green arrows); (E) coronal image depicting ascites (red x) and thickened sigmoid (green arrow).

Over the next 24 to 48 hours, she had continued diarrheal disease, abdominal pain, worsening of abdominal distension with shifting dullness, tachycardia, borderline low blood pressure, and fever, associated with worsening leukocytosis and acute kidney injury (Table 1). The ultrasound of the abdomen also reported ascites but no evidence of chronic liver disease, portal hypertension, or hepatoportal venous thrombosis. A computed tomography (CT) of the abdomen and pelvis revealed extensive colitis, ascites, and mild bilateral pleural effusion; no evidence of megacolon, perforation, focal abscess(es), or mass lesion or cirrhosis (Figure 2).

Paracentesis was performed under ultrasound guidance, and about 900 ml of straw-colored fluid was drained. The fluid analyses revealed high protein levels, low serum-ascites albumin gradient (SAAG) ascites, neutrophil-predominant leukocytosis, and normal levels of bilirubin, lipase, glucose, and triglycerides (Table 2).

**Table 2:** Summary of ascitic fluid analysis

Parameter	Results
Total leukocyte count	1818 cells/μL
Neutrophil (%; count)	81%; 1472 cells/μL
Red blood cell count	214 cells/μL
Protein	2.6 g/L
Albumin	1.4 g/L
Glucose	98 mg/dL
Amylase	49 IU/L
Lactate dehydrogenase	272 IU/L
Adenosine deaminase	11.5 IU/L
Culture	Sterile

**Figure 3:**
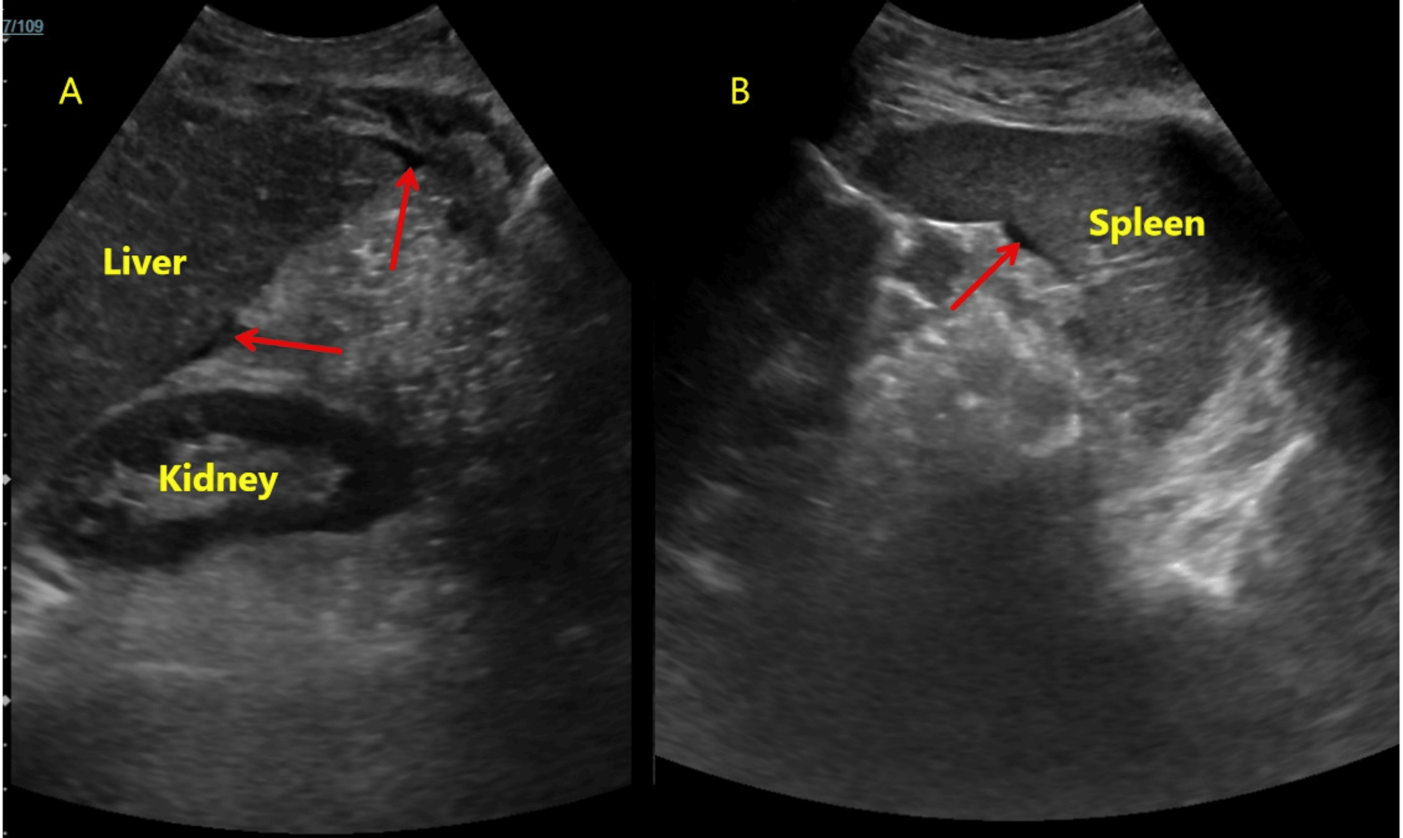
Ultrasound images Minimal fluid was demonstrated in the right (A) and left (B) upper quadrant sonographic images, with no evidence of cirrhosis (red arrows).

Two sets of ascitic fluid cultures, one in a sterile container and the other in blood culture bottles, reported no growth. Intravenous metronidazole was also added to the treatment regimen while oral vancomycin and hydration were continued. She responded to medical management, showing improvement in diarrhea, abdominal pain and distension, leukocyte count, and renal function within a week. A follow-up ultrasound revealed a marked reduction in ascites, which was not amenable to paracentesis for reassessment, and again showed no evidence of cirrhosis (Figure 3).

The multi-biomarker test, FibroTest®, was performed to exclude hepatic fibrosis, which could be missed in cross-sectional imaging; however, it was also reported as negative. The patient completed a 14-day course of intravenous metronidazole and oral vancomycin. She was discharged with a resolution of symptoms and remained stable at the one-month follow-up after discharge.

The differentials for colonic disease, in this case, include colonic neoplasm, inflammatory bowel disease (IBD), and infectious colitis. The patient did not undergo any endoscopic evaluation during the current hospitalization. However, she has had three screening colonoscopies to date, the last one eight months back: all reported normal except for hemorrhoids. Acute onset non-hemorrhagic diarrheal disease in patients with no relevant bowel diseases in the past and normal colonoscopies to date makes neoplasm and IBD unlikely diagnoses. An extensive stool analysis failed to detect any pathogen other than CD, excluding other infectious colitis.

## Discussion

The CD is ubiquitously distributed in nature, including in soil, sewage, animal excreta, and even in water and vegetables [9]. CD has been isolated from 7% to 15% of healthy individuals; these individuals may be colonizers or asymptomatic carriers, serving as reservoirs for the bacteria [10]. The CD spores, which are highly resistant to heat, desiccation, and many disinfectants and capable of surviving in the environment for a prolonged duration, are the infective form; oral ingestion serves as the primary portal of entry. The sources of infection include a contaminated hospital environment, equipment, and appliances, as well as healthcare personnel, including contaminated hands and stethoscopes, and, to a lesser extent, patient-to-patient transmission in healthcare facilities [11]. Under conducive conditions, the spores germinate, forming the vegetative form and producing toxins, which leads to infection [12]. The use of antimicrobial agents is the single most important predisposing factor for CD infection, as it alters the native gut microbiome [12]. The primary bile acids that promote CD spore germination are promptly deconjugated to secondary ones that are inhibitors of germination by the healthy gut microbiome. Loss of natural flora hence promotes CD spore germination.

Additionally, perturbed flora increases gut proline, succinate, and free sialic acid levels, all of which have been shown to promote CD infection. The depletion of natural flora reduces competition for nutrients, diminishes the inhibitory effects of innate flora, and again promotes pathogenic bacteria, including CD [12]. The concept of polymicrobial synergy often explains the increased risk of CD in hospitalized patients or those with multiple concomitant comorbidities. The simultaneous presence of multiple pathogens or inflammatory conditions leads to altered host immune responses, intermicrobial signaling, and metabolic sharing, promoting CD proliferation and infection [12]. Overall, a battery of postulated mechanisms, many yet to be proven, and likely many more unknown mechanisms, shift the innocuous CD at health to a deadly pathogen when sick or predisposed.

CD colitis typically presents as frequent, watery diarrhea. The presence of overt blood in stools is uncommon; however, patients may experience abdominal pain or cramps, fever, and signs of dehydration and electrolyte disturbances. Severe or fulminant CD colitis could lead to colonic ileus or toxic megacolon, with no diarrhea but abdominal distension, pain, and constipation. Colonic perforation, intra-abdominal abscesses, and systemic inflammatory response syndrome have been reported with severe infection. Extensive disease can result in protein-losing enteropathy, hypoalbuminemia, and ascites [13]. Clinically evident ascites are rare in CD; however, CT scans have reported an incidence of about 50% in patients with severe infection. The postulated mechanisms include hypoalbuminemia, transmural colonic inflammation, micro-perforation, and an inflammatory response or toxin-related increase in vascular permeability, leading to peritonitis [8,14]. CD peritonitis has been reported following GI perforation in CD colitis and sans perforation in patients on peritoneal dialysis or with concomitant cirrhosis [15,16]. Culture-negative, low SAAG, neutrophilic ascites complicating CD colitis, in the absence of perforation, concomitant cirrhosis, or peritoneal dialysis, is exceedingly rare, with only a few dozen cases published in the medical literature to date [8,14,17-19]. Interestingly, almost all of these culture-negative inflammatory ascites resolved after successful treatment of associated CD colitis. Zuckerman et al. postulates that this phenomenon is most likely the result of a toxin- or inflammation-mediated increase in vascular permeability or, to a lesser extent, the transmural migration of toxins or bacteria [17]. Only in a minority of cases was the CD toxin isolated from the peritoneal fluid, while culture remained negative in all cases. There are no specific guidelines on the treatment of inflammatory ascites in the setting of CD; hence, treatment follows the recommendations for CD colitis. The most recent guidelines recommend fidaxomicin as the first line, but vancomycin is considered an acceptable alternative. However, due to the cost, fidaxomicin therapy is sometimes restricted by budgetary concerns. In fulminant CD, a combination of oral vancomycin is the preferred agent, while the presence of ileus might require rectal vancomycin in combination with intravenous metronidazole [20,21].

Our patient, a middle-aged female with a history of chronic obstructive pulmonary disease, hypothyroidism, and recent antibiotic therapy for suspected pneumonia, presented with opiate overdose and developed severe CD colitis complicated by grade 2, low SAAG, neutrophil-predominant, culture-negative ascites in the absence of cirrhosis or overt perforation. The abdominal imaging reported ascites without any evidence of hepatic or renal diseases or overt visceral perforation but with diffuse thickening of the left colon suggestive of colitis. Hypoalbuminemia from protein-losing enteropathy may have contributed to ascites but is unlikely to be the sole cause, given the inflammatory nature of ascites. The mechanism of ascites is likely either a transmural inflammation or a toxic/inflammatory increase in intra-abdominal vascular permeability. Overt perforation was ruled out, but micro-perforation(s) is a possibility; generally, they tend to be culture-positive, growing polymicrobial enteric flora. We were unable to analyze the ascitic fluid for CD toxins, as the finding of inflammatory ascites was unexpected during the initial tap, and a repeat sampling was not possible due to the resolution of ascites, allowing no safe window. The medical management included oral vancomycin in combination with intravenous metronidazole for a total duration of 14 days. The diarrheal disease and ascites resolved with antibiotic therapy, and the patient was discharged without additional sequelae. Based on the current study and similar published case reports, it would be advisable to evaluate for ascites, especially in the setting of severe CD infection. Exclusion of perforation should continue to be the primary priority in cases of ascites associated with severe colitis, although ascites are increasingly reported in the absence of perforation. Furthermore, increased data availability could prompt further research into the etiopathogenesis, evaluation, and specific management recommendations in the setting of ascites in CD infection.

## Conclusions

Ascites is an uncommon but increasingly recognized manifestation of severe CD infection and could result in hypoalbuminemia, transmural inflammation, toxin-mediated capillary leak, or perforation. In the absence of alternative causes or perforation, treatment adhering to protocols for colonic CD infection, including pharmacotherapy with oral vancomycin with or without intravenous metronidazole, is efficient in managing this rare sequela.
